# Differences Between Habitual Low and Normal Carbohydrate Intake in Body Composition and Maximal Isometric Strength in Resistance-Trained Men

**DOI:** 10.3390/jfmk11030351

**Published:** 2026-09-03

**Authors:** Sergio Añasco Saca, Lilia Soifer Velásquez, Alexander Javier Iman Torres, Michelle Lozada-Urbano, Delsi M. Huaita Acha, Antonio Castillo-Paredes, José Antonio Pino Arango, Jose Jairo Narrea Vargas

**Affiliations:** 1 Grupo de Investigación en Nutrición, Metabolismo y Ejercicio, Facultad de Ciencias de la Salud, Carrera de Nutrición y Dietética, Universidad Científica del Sur, Lima 15067, Peru; 100083694@cientifica.edu.pe (S.A.S.); 100090417@cientifica.edu.pe (L.S.V.); 2 Departamento de Ciencia y Tecnología de Alimentos, Facultad de Industrias Alimentarias, Universidad Nacional de la Amazonía Peruana, Iquitos 16001, Peru; alexander.iman@unapiquitos.edu.pe; 3 Programa Académico en Nutrición y Dietética, Universidad Privada Norbert Wiener, Lima 15046, Peru; michelle.lozada@uwiener.edu.pe; 4 Dirección General de Investigaciones, Universidad Inca Garcilaso de la Vega, Lima 15084, Peru; delsi.huaita@uigv.edu.pe (D.M.H.A.); jose.pino@uigv.edu.pe (J.A.P.A.); 5 Grupo AFySE, Investigación en Actividad Física y Salud Escolar, Escuela de Pedagogía en Educación Física, Facultad de Educación, Universidad de Las Américas, Santiago 8370040, Chile; acastillop85@gmail.com

**Keywords:** dietary carbohydrates, body composition, resistance training, muscle strength dynamometer

## Abstract

**Introduction:** Most studies evaluating low-carbohydrate diets in resistance-trained individuals have focused on short-term ketogenic interventions. Whether habitual carbohydrate intake maintained under free-living conditions is associated with body composition and muscle strength remains unclear. Therefore, this study compared body composition and maximal isometric strength between recreationally resistance-trained men with a habitual low or normal carbohydrate intake. **Methods:** A cross-sectional analytical study was conducted in 53 recreationally resistance-trained men aged 18–35 years. Habitual dietary intake was assessed using three non-consecutive 24 h dietary recalls. Participants were classified as having low (<3 g/kg/day; *n* = 28) or normal (3–5 g/kg/day; *n* = 25) carbohydrate intake; participants also reported maintaining their respective dietary pattern for at least three months. Body composition was estimated using the Ross and Kerr anthropometric model, whereas fat mass was calculated using the Martin equation. Maximal isometric handgrip and knee extension strength were also assessed. Between-group comparisons were performed using Welch’s *t*-test or the Mann–Whitney U test, and multiple linear regression models were adjusted for age, height, and relative energy intake. Sensitivity analyses were additionally adjusted for relative protein intake. **Results:** Participants with a habitual low carbohydrate intake presented significantly lower adipose mass (β = −2.42 kg; 95% CI: −4.49 to −0.35; *p* = 0.02), adipose percentage (β = −4.64%; 95% CI: −7.47 to −1.80; *p* < 0.01), fat mass (β = −2.33 kg; 95% CI: −4.08 to −0.57; *p* = 0.01), and body fat percentage (β = −4.06%; 95% CI: −6.52 to −1.59; *p* < 0.01) than participants with a normal carbohydrate intake. No significant differences were observed in muscle mass, handgrip strength, or absolute knee extension strength after adjustment. In sensitivity analyses additionally adjusted for relative protein intake, associations with adiposity were attenuated and no longer statistically significant, whereas low carbohydrate intake was associated with lower absolute and relative handgrip and knee extension strength (all *p* < 0.05). No significant associations with skeletal muscle mass were observed. **Conclusions:** In recreationally resistance-trained men, habitual carbohydrate intake below 3 g/kg/day was associated with lower adiposity in the primary analyses; however, these associations were attenuated after additional adjustment for relative protein intake. Conversely, associations with lower maximal isometric strength emerged after adjustment for protein intake, while no associations with skeletal muscle mass were observed. These findings suggest that the associations of habitual carbohydrate intake with adiposity and muscle strength may be influenced by overall dietary composition and should be interpreted cautiously given the cross-sectional design.

## 1. Introduction

Carbohydrate intake is one of the main nutritional determinants of training adaptations and athletic performance [1]. During moderate- and high-intensity exercise, carbohydrates serve as the primary substrate for ATP resynthesis through glycolysis and contribute to the maintenance of muscle glycogen stores, thereby sustaining force production, training volume, and recovery between training sessions [2,3]. Consequently, current sports nutrition guidelines emphasize that daily carbohydrate intake should be adjusted according to training demands, ensuring adequate carbohydrate availability to optimize performance and exercise-induced adaptations [3].

In resistance training, the role of carbohydrates has traditionally received less attention than protein intake. Nevertheless, resistance exercise relies heavily on glycolytic metabolism, particularly when multiple sets, high training volumes, or exercises performed at moderate to high intensities are involved [4,5]. Under these conditions, the progressive depletion of muscle glycogen may impair the ability to maintain performance throughout the training session and reduce the cumulative mechanical stimulus, one of the primary drivers of muscular adaptations [2,6].

Beyond their role as an energy substrate, carbohydrate availability also appears to influence several cellular processes involved in training adaptation [7]. Recent evidence suggests that a low carbohydrate intake may increase the oxidation of branched-chain amino acids, reduce the availability of essential amino acids required for muscle protein synthesis, and attenuate the expression of key regulatory factors involved in myogenesis. When sustained over several weeks, these alterations in anabolic signaling may limit resistance training-induced muscle hypertrophy and impair performance during high-intensity anaerobic exercise [8].

Consistent with these physiological mechanisms, current recommendations for individuals performing resistance training suggest a daily carbohydrate intake of approximately 3–7 g/kg/day, adjusted according to training volume, intensity, and frequency [2]. Likewise, the recent position stand of the International Society of Sports Nutrition acknowledges that diets with marked carbohydrate restriction may represent a useful strategy in specific contexts, particularly for reducing fat mass. However, it also concludes that current evidence remains insufficient to support these dietary approaches as superior when the primary goal is to maximize resistance training adaptations, including skeletal muscle hypertrophy and neuromuscular performance [9].

Despite the growing interest in carbohydrate-restricted dietary strategies, most available evidence derives from short-term experimental interventions, primarily comparing ketogenic diets with conventional diets. Although these studies have improved our understanding of the physiological effects of severe carbohydrate restriction, they provide limited information regarding habitual carbohydrate intake, defined as the daily carbohydrate consumption consistently maintained over prolonged periods under habitual training conditions. This distinction is clinically and practically relevant because many recreationally trained individuals consume carbohydrate amounts below current recommendations without adhering to a strict ketogenic diet, potentially resulting in chronically reduced carbohydrate availability.

Consequently, a knowledge gap remains regarding whether lower habitual carbohydrate intake is associated with differences in body composition and strength performance among resistance-trained adult men. Evidence addressing this question may help determine whether adherence to current carbohydrate intake recommendations is associated with morphological and functional outcomes relevant to individuals engaged in resistance training.

Therefore, the aim of the present study was to compare body composition and isometric strength performance between resistance-trained adult men with low habitual carbohydrate intake and those with normal habitual carbohydrate intake. We hypothesized that participants with lower habitual carbohydrate intake would exhibit lower fat mass percentage and absolute fat mass, as well as differences in other body composition indicators and strength performance compared with those with normal habitual carbohydrate intake.

## 2. Materials and Methods

### 2.1. Study Design

A cross-sectional analytical observational study was conducted to compare body composition and maximal isometric strength between two independent groups of recreationally resistance-trained men classified according to their habitual carbohydrate intake (low versus normal carbohydrate intake). The study did not involve any dietary or training intervention. Body composition and strength assessments were performed during a single study visit, whereas habitual dietary intake was estimated using three 24 h dietary recalls administered during the same week. The study was reported in accordance with the Strengthening the Reporting of Observational Studies in Epidemiology (STROBE) statement for cross-sectional studies [10].

### 2.2. Participants

Participants were recruited using a non-probability convenience sampling approach from regular members of a gym located in the Los Olivos district of Metropolitan Lima, Peru. Only men with previous resistance training experience were included to compare body composition and strength performance between individuals with different levels of habitual carbohydrate intake while minimizing the influence of early training adaptations.

Participants were eligible if they met the following inclusion criteria: being registered at the gym where the assessments were conducted for at least three months, allowing verification of training attendance; being between 18 and 35 years of age; being male; and having at least one year of continuous resistance training experience, defined as a minimum frequency of two training sessions per week (or eight non-consecutive sessions per month) without interruptions longer than 30 consecutive days. Resistance training experience during the preceding year was determined by participant self-report, whereas gym attendance records were used only to verify recent attendance during the period for which records were available at the study site.

Habitual carbohydrate intake was subsequently estimated using three 24 h dietary recalls. Based on this assessment, participants were classified according to their habitual carbohydrate intake, and only those with low or normal carbohydrate intake were included in the analyses. Participants with a habitual carbohydrate intake exceeding 5 g/kg/day were excluded. The classification of habitual carbohydrate intake is described in the Dietary assessment section.

Participants were also excluded if they had physician-diagnosed conditions that could affect body composition or physical performance, including diabetes mellitus, liver disease, kidney disease, or cancer; if they were using medications with potential effects on body composition or muscle strength, including metformin, corticosteroids, diuretics, sodium–glucose cotransporter-2 (SGLT-2) inhibitors, or glucagon-like peptide-1 (GLP-1) receptor agonists; or if they reported the use of anabolic–androgenic steroids during the previous 12 months. This information was obtained through a confidential interview conducted by the investigators (S.A.S. and L.S.V.) during the initial assessment.

### 2.3. Sample Size

The sample size was calculated to detect differences in body fat mass, considered the primary outcome of the study, between participants with low and normal habitual carbohydrate intake. Because no observational studies had reported differences in this outcome according to habitual carbohydrate intake, the effect size was estimated from an experimental study conducted by Vidić et al. [11], which compared body composition between resistance-trained individuals following either a ketogenic diet or a conventional diet. Based on the data reported in that study, a Cohen’s d effect size of 1.06 was estimated. We acknowledge that this represents a large effect size derived from an experimental dietary intervention and may not directly reflect the magnitude of between-group differences expected in the present observational setting.

The sample size was calculated using G*Power version 3.1.9.7 (Heinrich Heine University Düsseldorf, Düsseldorf, Germany) based on a two-tailed independent-samples t test, assuming a significance level of α = 0.05, a statistical power of 80%, and an allocation ratio of 1:1. Under these assumptions, the minimum required sample size was estimated at 15 participants per group (30 participants in total).

**Figure 1:**
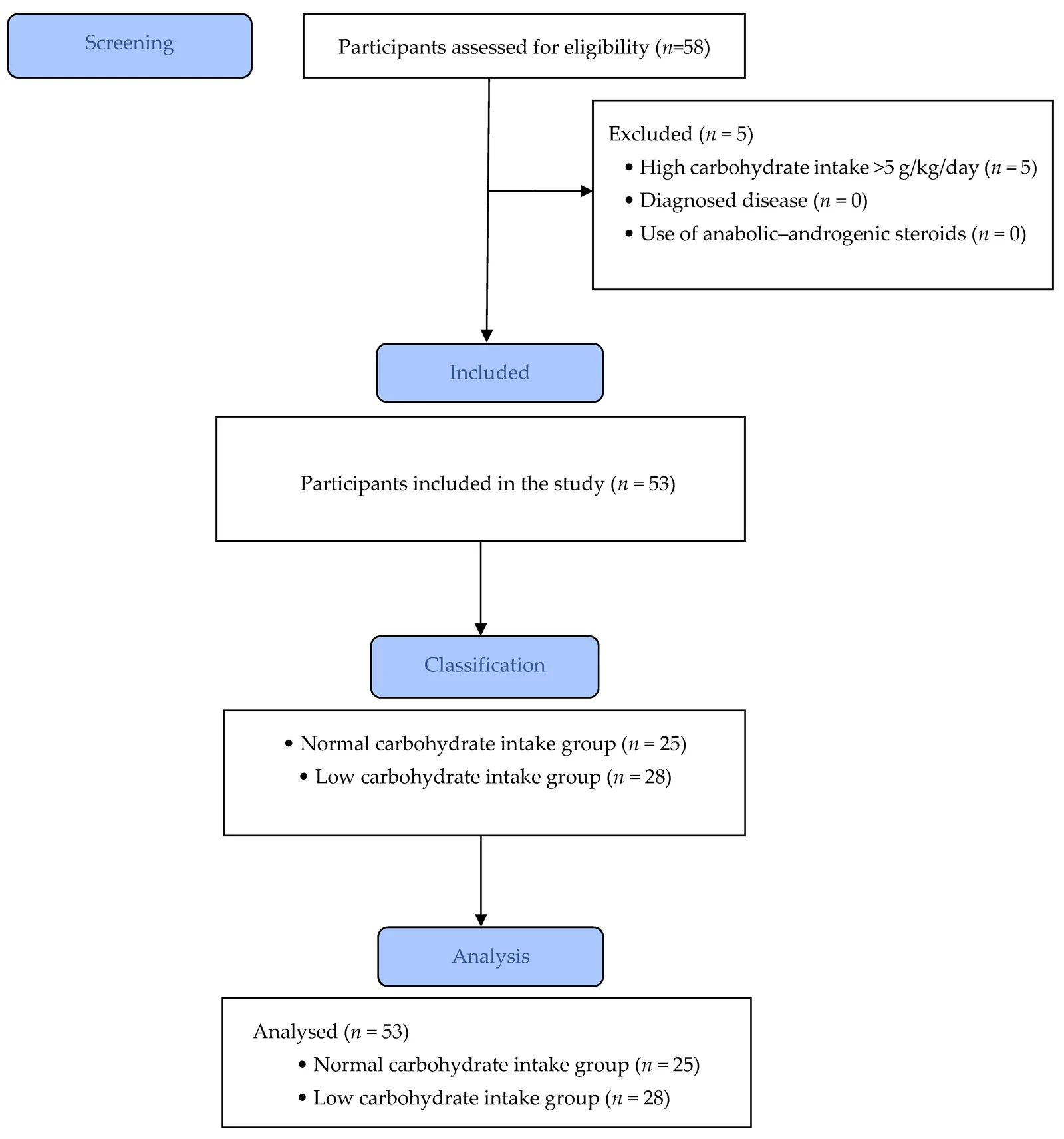
Flow diagram of participant selection and inclusion in the study.

The calculated sample size represented the minimum number of participants required rather than a prespecified maximum sample size. Recruitment continued throughout the predefined recruitment period from May to July 2026, and all participants who met the eligibility criteria during this period were included. No interim analyses or data-dependent stopping rules were applied. Consequently, the final sample comprised 53 resistance-trained men, of whom 28 were classified as having low habitual carbohydrate intake and 25 as having normal habitual carbohydrate intake, exceeding the minimum sample size required by the a priori calculation. The participant recruitment, selection, and classification process is shown in Figure 1. A sensitivity analysis indicated that this sample provided 80% statistical power to detect an approximate Cohen’s d effect size of 0.79.

### 2.4. Procedures

Participant recruitment and study assessments were conducted between May and July 2026 at a gym located in the Los Olivos district of Metropolitan Lima, Peru. Participants were recruited on-site by inviting regular gym members to voluntarily participate in the study.

Because of participants’ availability, each participant was assessed on a different day following a standardized protocol. First, the study objectives were explained, and written informed consent was obtained. Subsequently, the first 24 h dietary recall was administered through an individual interview, followed by the body composition assessment and the measurement of maximal isometric strength. These three assessments were completed during a single study visit lasting approximately 60 min. All procedures were performed by the same investigators (S.A.S. and L.S.V.), who had received prior training and followed standardized protocols to minimize inter-rater variability.

#### 2.4.1. Dietary Assessment

Dietary intake was assessed using three retrospective 24 h dietary recalls (24HRs) administered according to the multiple-pass method, a structured interview technique consisting of several sequential steps designed to facilitate comprehensive recall and recording of all foods and beverages consumed during the previous 24 h. This method has demonstrated adequate validity and reproducibility for assessing dietary intake in adults [12].

The three 24HRs were administered on non-consecutive days within the same week, including at least one weekend day or public holiday, to obtain a more representative estimate of participants’ habitual dietary intake. The first interview was conducted face-to-face in a dedicated room within the gym, whereas the second and third interviews were conducted via videoconference using Google Meet (Google LLC, Mountain View, CA, USA), according to each participant’s availability. Each interview lasted approximately 20 min. To improve the estimation of portion sizes, a photographic food atlas was used as a visual aid [13].

The nutrient composition of the reported foods was determined using the Peruvian Food Composition Tables [14], from which daily energy intake (kcal/day) and macronutrient intake (g/day) were calculated. Subsequently, carbohydrate, protein, and fat intake were expressed relative to body weight (g/kg/day) using the body weight measured during the anthropometric assessment. The mean daily intake of energy and each macronutrient was then calculated from the three 24HRs.

Finally, participants were classified according to the mean carbohydrate intake estimated from the three 24HRs, provided that they self-reported maintaining a comparable dietary pattern for at least three months. These cut-off values were established based on current sports nutrition recommendations [2,3]. Although carbohydrate requirements for resistance training may range from approximately 3 to 7 g/kg/day depending on training volume, intensity, and performance goals [2], recommendations for individuals participating in general fitness programs indicate that a normal carbohydrate intake of approximately 45–55% of total energy, corresponding to 3–5 g/kg/day, is generally sufficient to meet daily carbohydrate needs [3]. Given that the participants in the present study were recreationally resistance-trained individuals rather than competitive athletes following high-volume performance-oriented programs, an intake of 3–5 g/kg/day was therefore classified as normal habitual carbohydrate intake, whereas intake below 3 g/kg/day was classified as low habitual carbohydrate intake.

#### 2.4.2. Body Composition

Anthropometric and body composition assessments were performed in a private, dedicated room within the gym facilities. All assessments were conducted in the afternoon during the participants’ usual attendance period. Participants were assessed at least three hours after their last main meal and before performing their resistance training session on the assessment day. Thus, no exercise was performed immediately before the measurements, and assessments were not conducted in a fasted state. Before the assessment, participants were given approximately 10 min to prepare and were evaluated wearing minimal clothing, following international recommendations to minimize measurement error.

Anthropometric measurements were performed according to the International Society for the Advancement of Kinanthropometry (ISAK) protocol [15]. Body weight was measured with participants barefoot and wearing light clothing using a digital scale (Omron Karada Scan HBF-701; Omron Healthcare Co., Ltd., Kyoto, Japan) with an accuracy of ±1%. Standing height was measured using a stadiometer certified by the National Center for Food, Nutrition and Healthy Living (CENAN, Lima, Peru), with a precision of 0.1 cm.

Skinfold thicknesses were measured using a Slim Guide skinfold caliper (Creative Health Products Inc., Plymouth, MI, USA) with a precision of 1 mm, whereas body circumferences were measured using an anthropometric tape (AVA-04; Avanutri Equipamentos de Saúde Ltd.a., Rio de Janeiro, Brazil) with a precision of 0.1 cm.

All anthropometric measurements were performed by the same anthropometrist, who had received specific training in ISAK measurement procedures but was not formally ISAK-certified. Measurement quality was evaluated through technical error of measurement (TEM), which was <5% for skinfold thicknesses and <1.5% for all other anthropometric measurements. Skinfold thicknesses were measured at the triceps, subscapular, supraspinale, abdominal, medial thigh, and medial calf sites. Circumference measurements included the relaxed upper arm, forearm, chest, thigh, and calf.

Adipose mass and skeletal muscle mass were estimated using the body mass fractionation method proposed by Kerr [16], which applies the Phantom anthropometric reference model to standardize body dimensions and estimate the different body composition components. Fat mass was subsequently estimated from adipose mass using the equation proposed by Martin et al. [17], which accounts for variations in the lipid fraction of adipose tissue according to the degree of adiposity. Finally, the percentage of each body composition component was calculated by dividing the estimated mass of each component by body weight and multiplying the result by 100.

#### 2.4.3. Maximal Isometric Strength

Maximal isometric strength was assessed immediately after the anthropometric evaluation and before each participant’s habitual training session. Isometric strength assessments were selected primarily because of their feasibility, accessibility, and ease of standardization under field conditions, allowing both upper- and lower-body maximal force to be evaluated using portable dynamometry without requiring specialized laboratory equipment.

Before testing, all participants completed a standardized warm-up consisting of one set of 10 repetitions of dumbbell biceps curls and one set of 10 repetitions of machine knee extensions, using a load corresponding to a rating of perceived exertion (RPE) of 5–6 on the Borg CR-10 scale. A 60 s recovery period was provided between the two exercises before the strength assessments.

Maximal isometric knee extension strength was assessed using an E-lastic dynamometer (Brasília, Brazil) with a maximum capacity of 200 kg and a precision of 0.1 kg. During the test, participants were seated on a clinical examination table with the hip and knee of the dominant leg flexed at 90°. The dynamometer was secured to the examination table using a non-elastic chain and positioned around the distal portion of the lower leg, immediately above the ankle, allowing participants to perform an isometric contraction against an immovable resistance. Participants were then instructed to perform a maximal knee extension by gradually increasing force until reaching maximal voluntary contraction [18].

Maximal isometric handgrip strength was assessed in the dominant hand using a Baseline hydraulic hand dynamometer (Fabrication Enterprises Inc., White Plains, NY, USA) with a maximum capacity of 90.7 kg and a precision of 2 kg. The test was performed with participants standing upright, with the dominant arm fully extended alongside the body. Participants were instructed to squeeze the dynamometer handle with maximal effort for five seconds [19].

For both strength tests, three maximal trials were performed. Each contraction was maintained for up to five seconds, with a recovery period of 120–180 s between trials to minimize the effects of fatigue. The highest recorded value was considered the participant’s maximal isometric strength. Strength was subsequently expressed in both absolute (kg) and relative (kg/kg body weight) terms, with relative strength calculated by dividing absolute strength (kg) by body weight (kg).

### 2.5. Ethical Considerations

The study was approved by the Institutional Research Ethics Committee of Universidad Científica del Sur (approval code: 1377-CIEI-CIENTIFICA-2025; approval date: 3 December 2025). All study procedures were conducted in accordance with the ethical principles of the Declaration of Helsinki [20]. Written informed consent was obtained from all participants before the initiation of any study procedures. Participant confidentiality was protected by coding personal identifiers and restricting access to the study database, which was managed in accordance with the Peruvian Personal Data Protection Law (Law No. 29733) and its implementing regulations.

### 2.6. Statistical Analysis

Statistical analyses were performed using IBM SPSS Statistics version 27.0 (IBM Corp., Armonk, NY, USA) and Jamovi version 2.7.38 (The jamovi Project, Sydney, Australia). The distribution of continuous variables was assessed using the Shapiro–Wilk test and by visual inspection of histograms and normal probability plots (Q–Q plots).

Normally distributed variables are presented as mean and standard deviation (SD), whereas non-normally distributed variables are reported as median and interquartile range (IQR). Categorical variables are presented as absolute frequencies and percentages.

Comparisons between the normal and low habitual carbohydrate intake groups were performed using Welch’s t test for normally distributed variables and the Mann–Whitney U test for non-normally distributed variables. The categorical variable was compared using Fisher’s exact test. For variables analyzed using Welch’s t test, the mean difference, its 95% confidence interval (95% CI), and Cohen’s d were reported as measures of effect size. Cohen’s d values were interpreted as trivial (<0.20), small (0.20–0.49), moderate (0.50–0.79), and large (≥0.80). For variables analyzed using the Mann–Whitney U test, between-group differences were estimated using the Hodges–Lehmann estimator with 95% CI, and rank-biserial correlation (rrb) was reported as the effect size.

Subsequently, independent multiple linear regression models were constructed to estimate the adjusted differences in each body composition and muscle strength variable according to habitual carbohydrate intake. The independent variable was carbohydrate intake group, coded as 0 = normal carbohydrate intake and 1 = low carbohydrate intake. Each body composition and muscle strength indicator was analyzed as the dependent variable in a separate model. All models were adjusted for age, height, and relative energy intake (kcal/kg/day). Relative strength variables were calculated as simple ratios of absolute strength to body weight (kg/kg). Body weight was not included as an additional covariate in regression models for relative strength outcomes to avoid redundant adjustment and potential multicollinearity.

The assumptions of linear regression, including linearity, homoscedasticity, normality of residuals, and absence of multicollinearity, were assessed before model estimation and interpretation. Multicollinearity was evaluated using the variance inflation factor (VIF) and tolerance. Results are presented as adjusted between-group differences (unstandardized β coefficients), together with their 95% confidence intervals (95% CI), *p* values, and adjusted coefficients of determination (adjusted R^2^).

Given the significant between-group difference in relative protein intake and its potential influence on body composition and muscle strength, a sensitivity analysis was additionally performed by including relative protein intake (g/kg/day) as a covariate in each of the regression models. Multicollinearity was reassessed after inclusion of relative protein intake using VIF and tolerance values. The results of these sensitivity analyses are presented in Supplementary Table S1.

Adjusted differences from the primary regression models were graphically displayed using forest plots created with the ggplot2 and patchwork packages in R version 4.5.0 (R Foundation for Statistical Computing, Vienna, Austria). Fat mass was considered the primary outcome, as it was the outcome used for the a priori sample size calculation, whereas the remaining body composition and muscle strength variables were considered secondary outcomes. No formal adjustment for multiple comparisons was applied because these secondary outcomes were physiologically related and intended to provide complementary information on body composition and muscle strength rather than to represent independent confirmatory hypotheses. Nevertheless, because multiple statistical tests were performed, findings for secondary outcomes were interpreted cautiously in view of the increased risk of type I error. Statistical significance was set at a two-sided *p* < 0.05.

## 3. Results

A total of 53 recreationally resistance-trained men completed the study assessments, of whom 25 were classified as having normal habitual carbohydrate intake and 28 as having low habitual carbohydrate intake. As shown in Table 1, the two groups were comparable with respect to age, body weight, height, and body mass index (all *p* > 0.05). Regarding dietary intake, the normal carbohydrate intake group exhibited a higher relative energy intake than the low carbohydrate intake group (31.5 ± 3.3 vs. 25.5 ± 1.7 kcal/kg/day; *p* < 0.001), whereas the low carbohydrate intake group had a higher protein intake (1.7 [1.6–1.7] vs. 1.4 [1.4–1.6] g/kg/day; *p* < 0.001). No significant between-group differences were observed in fat intake or training frequency (all *p* > 0.05).

Body composition and muscle strength according to habitual carbohydrate intake are presented in Table 2. Participants with normal habitual carbohydrate intake exhibited higher adipose mass (17.17 ± 3.00 vs. 14.75 ± 2.44 kg; *p* < 0.01), adipose mass percentage (23.70 ± 3.74 vs. 20.45 ± 2.71%; *p* < 0.001), fat mass (10.76 ± 2.49 vs. 8.64 ± 1.83 kg; *p* = 0.001), and body fat percentage (14.87 ± 3.33 vs. 11.97 ± 2.26%; *p* < 0.001) than participants with low habitual carbohydrate intake, with large effect sizes (Cohen’s d = 0.88–1.02). No significant between-group differences were observed for skeletal muscle mass, expressed in either absolute or relative terms (all *p* > 0.05). Likewise, no significant between-group differences were found in handgrip strength or isometric knee extension strength, expressed in either absolute or relative terms (all *p* > 0.05).

The results of the multiple linear regression models adjusted for age, height, and relative energy intake are presented in Table 3. Compared with the normal habitual carbohydrate intake group, participants with low habitual carbohydrate intake had 2.42 kg less adipose mass (β = −2.42; 95% CI: −4.49 to −0.35; *p* = 0.02), 4.64 percentage points lower adipose mass percentage (β = −4.64; 95% CI: −7.47 to −1.80; *p* < 0.01), 2.33 kg less fat mass (β = −2.33; 95% CI: −4.08 to −0.57; *p* = 0.01), and 4.06 percentage points lower body fat percentage (β = −4.06; 95% CI: −6.52 to −1.59; *p* < 0.01). No statistically significant between-group differences were observed for skeletal muscle mass, expressed in either absolute or relative terms, handgrip strength, expressed in either absolute or relative terms, or absolute isometric knee extension strength (all *p* > 0.05). However, participants with low habitual carbohydrate intake exhibited 0.041 kg/kg lower relative isometric knee extension strength than those with normal habitual carbohydrate intake (β = −0.041 kg/kg; 95% CI: −0.082 to −0.001; *p* = 0.04).

In sensitivity analyses additionally adjusted for relative protein intake, the associations between low habitual carbohydrate intake and adiposity outcomes were attenuated and were no longer statistically significant (all *p* > 0.05). Conversely, low habitual carbohydrate intake was associated with lower absolute and relative handgrip and knee extension strength (all *p* < 0.05). No significant associations were observed for skeletal muscle mass. No evidence of problematic multicollinearity was identified, with VIF values ranging from 1.079 to 3.357 and tolerance values ranging from 0.298 to 0.927. Full results are presented in Supplementary Table S1.

**Figure 2:**
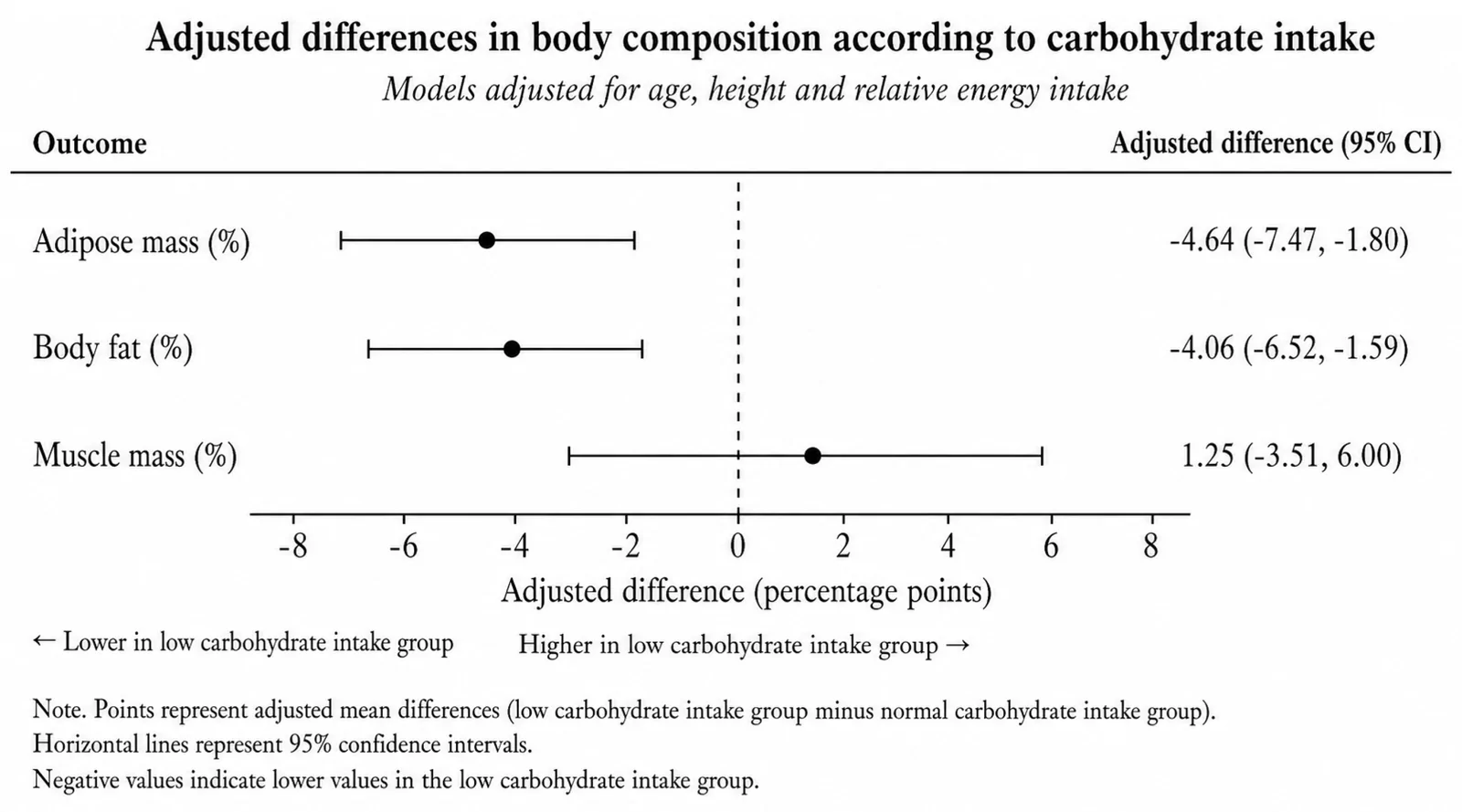
Adjusted differences in body composition according to habitual carbohydrate intake.

Figure 2 illustrates the adjusted differences in percentage-based body composition indicators, showing lower adipose mass percentage and body fat percentage in the low habitual carbohydrate intake group, whereas no difference was observed in skeletal muscle mass percentage.

## 4. Discussion

The present study compared body composition and muscle strength between recreationally resistance-trained adult men with low (<3 g/kg/day) or normal (3–5 g/kg/day) habitual carbohydrate intake. The main findings indicate that participants with low habitual carbohydrate intake exhibited lower adipose mass and fat mass, both in absolute and relative terms, whereas no significant differences were observed in skeletal muscle mass or maximal isometric handgrip and knee extension strength in the unadjusted comparisons. Furthermore, after adjustment for age, height, and relative energy intake, low habitual carbohydrate intake remained independently associated with lower adiposity, but not with skeletal muscle mass or most muscle strength outcomes. However, sensitivity analyses additionally adjusting for relative protein intake attenuated the associations with adiposity, which were no longer statistically significant, while associations emerged between low carbohydrate intake and lower absolute and relative handgrip and knee extension strength. Skeletal muscle mass remained unrelated to carbohydrate intake group. These findings indicate that the observed associations, particularly those involving adiposity and strength, are sensitive to adjustment for dietary composition and should therefore be interpreted cautiously.

Our findings are consistent with experimental evidence demonstrating that carbohydrate restriction may influence body composition in resistance-trained individuals. Ribeiro et al. [21] examined young resistance-trained men classified according to lower or higher habitual carbohydrate intake during an eight-week progressive resistance training program. Both groups increased lean soft tissue and muscular strength, with no significant between-group differences in these outcomes, although greater responsiveness in lean soft tissue and arm-curl strength was observed among participants with higher carbohydrate intake. Similarly, several randomized controlled trials conducted in resistance-trained individuals have shown that ketogenic or very-low-carbohydrate diets may reduce fat mass without substantially compromising fat-free mass [11,22,23]. These findings are further supported by the systematic review by Coleman et al. [24], which synthesized evidence from 13 studies involving physically active individuals and concluded that ketogenic diets consistently promote fat mass reduction while generally preserving fat-free mass, although considerable heterogeneity existed across studies. Although these findings are consistent with the lower adiposity observed in our primary models, the associations were attenuated and no longer statistically significant after additional adjustment for relative protein intake. Therefore, the observed differences in adiposity cannot be attributed to carbohydrate intake independently of other aspects of dietary composition.

However, an important methodological difference should be highlighted when comparing our findings with the existing literature. Whereas previous experimental studies have primarily evaluated short-term ketogenic interventions (typically lasting 8–12 weeks) designed to induce nutritional ketosis [11,22,23,25], the present study classified participants according to their habitual carbohydrate intake maintained for at least three months. Consequently, our findings provide complementary observational evidence regarding body composition characteristics associated with different reported levels of carbohydrate intake under free-living conditions.

A plausible physiological explanation is that lower carbohydrate intake induces metabolic adaptations that increase reliance on lipids as the primary energy source. Reduced muscle and liver glycogen availability promotes greater mobilization of fatty acids, lowers circulating insulin concentrations, and increases the activity of proteins involved in fatty acid transport and β-oxidation [8]. Consistent with these mechanisms, Coleman et al. [24] reported that very low-carbohydrate diets increase the availability of free fatty acids and enhance lipid oxidation, which may explain the reduction in fat mass observed in physically active individuals. Similarly, Cholewa et al. [6] suggested that adaptation to reduced carbohydrate availability promotes greater utilization of fat as a metabolic fuel, particularly after several weeks of dietary intervention. However, because muscle glycogen, circulating ketone bodies, and substrate oxidation rates were not assessed in the present study, these mechanisms should be regarded only as plausible biological explanations rather than processes directly demonstrated by our findings.

Another factor that likely contributed to our findings was the lower relative energy intake observed in the low carbohydrate intake group. Although this potential confounder was accounted for in the multivariable analyses, the influence of energy balance on body composition cannot be completely ruled out because of the cross-sectional design of the study. In this regard, Coleman et al. [24] proposed that much of the reduction in fat mass observed during ketogenic diets may be attributable to a spontaneous decrease in energy intake, even when experimental protocols were designed to maintain energy balance. Moreover, relative protein intake was significantly higher in the low carbohydrate intake group. When protein intake was additionally included in the sensitivity models, the associations between carbohydrate intake group and adiposity were attenuated and no longer statistically significant. This finding suggests that the lower adiposity observed in the primary models may partly reflect differences in the broader dietary pattern rather than carbohydrate intake alone. However, because carbohydrate, protein, and total energy intake are interrelated components of dietary intake, the sensitivity analysis should not be interpreted as establishing protein intake as the causal explanation for these differences.

In contrast to adiposity, no significant between-group differences were observed in skeletal muscle mass. This finding is consistent with the results reported by Ribeiro et al. [21] and several randomized controlled trials showing that lower carbohydrate intake does not necessarily compromise fat-free mass when protein intake is adequate and resistance training is maintained [11,22,23]. Likewise, Coleman et al. [24] concluded that the preservation of fat-free mass during low-carbohydrate diets may be explained, at least in part, by sufficient protein intake and the mechanical stimulus provided by resistance training. In the present study, both groups consumed protein amounts consistent with current recommendations for resistance-trained individuals [3], and protein intake was even slightly higher in the low carbohydrate intake group, which may have contributed to the maintenance of skeletal muscle mass. Importantly, the absence of an association between carbohydrate intake group and skeletal muscle mass persisted in the sensitivity analyses additionally adjusted for relative protein intake, suggesting that this finding was comparatively robust to adjustment for differences in protein consumption.

Nevertheless, it is important to note that some studies have reported greater gains in muscle mass with higher carbohydrate availability during hypertrophy-oriented resistance training programs. Both Vargas et al. [22] and Paoli et al. [23] observed significant increases in fat-free mass only in the groups with higher carbohydrate intake, whereas Borruel Abadía et al. [26] reported a more favorable hypertrophic response with a high-carbohydrate diet than with a ketogenic diet. These discrepancies likely reflect the greater importance of carbohydrate availability when the primary objective is to maximize hypertrophic adaptations induced by highly controlled resistance training programs.

Regarding muscle strength, the interpretation of our findings is less straightforward. In the primary models adjusted for age, height, and relative energy intake, no clear associations were observed between carbohydrate intake group and most maximal isometric strength outcomes. However, after additional adjustment for relative protein intake, participants with low habitual carbohydrate intake exhibited significantly lower absolute and relative handgrip and knee extension strength. This change in the estimates suggests that the relationship between carbohydrate intake and strength may be sensitive to differences in dietary composition. Given that protein intake differed significantly between groups and the sensitivity models included an additional covariate in a relatively small sample, these findings should be regarded as exploratory and interpreted cautiously rather than as evidence of an independent detrimental effect of lower carbohydrate intake on strength. The results of the primary models are consistent with those reported by Vidić et al. [11], Paoli et al. [23], and Wilson et al. [27], who observed no significant differences in maximal strength between ketogenic and conventional diets despite differences in body composition. In contrast, Borruel Abadía et al. [26] reported greater improvements in maximal strength performance following a high-carbohydrate diet, although both groups improved after the training intervention. Interestingly, the direction of the associations observed in our protein-adjusted sensitivity analyses is more consistent with the latter finding, although direct comparisons are limited by substantial differences in study design, carbohydrate exposure, and strength assessment. These discrepancies may be explained by differences in intervention duration, training volume, the degree of carbohydrate restriction, and the type of strength assessment used. Furthermore, Vargas-Molina et al. [25] observed that participants experienced higher ratings of perceived exertion and a slight reduction in training volume during the first weeks of a ketogenic diet. However, these differences disappeared as the intervention progressed, suggesting that the initial effects of carbohydrate restriction on exercise performance may be attenuated following metabolic adaptation.

From a physiological perspective, short-duration maximal isometric contractions rely primarily on the adenosine triphosphate–phosphocreatine (ATP-PCr) energy system, whereas muscle glycogen availability becomes increasingly important during prolonged, repeated, or high-volume exercise [8]. Therefore, the sensitivity of the observed associations with maximal isometric strength to model specification should not be interpreted as evidence that carbohydrate availability has no influence on other dimensions of resistance exercise performance. Dynamic strength, muscular endurance, repeated-set performance, and power output may be more sensitive to reduced carbohydrate availability because of their greater reliance on glycolytic metabolism and muscle glycogen. Consistent with this concept, the International Society of Sports Nutrition recommends maintaining high carbohydrate availability particularly during periods of prolonged exercise, multiple daily training sessions, or high training volumes [3], conditions that differ substantially from those evaluated in the present study.

### 4.1. Practical Implications

From a practical perspective, the primary analyses indicated that a habitual carbohydrate intake of <3 g/kg/day was associated with lower adiposity in young men engaged in recreational resistance training, without clear differences in skeletal muscle mass. However, these associations with adiposity were attenuated after additional adjustment for relative protein intake, while associations with lower maximal isometric strength emerged. Therefore, the present findings do not provide sufficient evidence to conclude that consuming <3 g/kg/day independently favors body composition without potential implications for strength. These findings should not be interpreted as a recommendation to systematically reduce carbohydrate intake, as the present study is observational and does not allow causal inferences to be drawn. Furthermore, based on the available experimental evidence, reduced carbohydrate availability may not represent the most appropriate strategy when the primary objective is to maximize muscle hypertrophy during resistance training programs [22,23].

In addition, expressing carbohydrate intake relative to body weight (g/kg/day) allowed the identification of differences in body composition between groups, supporting the practical utility of this approach for assessing and individualizing carbohydrate intake according to body weight rather than relying exclusively on the percentage of total energy derived from carbohydrates. Importantly, the 3–5 g/kg/day range used to define normal intake in the present study represents the lower portion of the broader 3–7 g/kg/day range suggested for resistance training and is particularly applicable to individuals engaged in general fitness or recreational training [2,3]. Higher carbohydrate intakes within this broader range may be warranted as training volume, intensity, frequency, or performance demands increase. Therefore, our findings should not be interpreted as indicating that 3–5 g/kg/day is optimal for all resistance-trained individuals. Consequently, carbohydrate intake should be individualized according to training volume, intensity, frequency, and the specific goals of each athlete [3].

### 4.2. Strengths and Limitations

A major strength of the present study is the evaluation of individuals who reported maintaining relatively stable carbohydrate intake patterns for at least three months, providing an opportunity to examine the associations between different levels of carbohydrate intake and body composition and muscle strength under free-living conditions. This approach differs from that of most available experimental studies, which have primarily evaluated short-term dietary interventions under highly controlled conditions. Consequently, our findings provide complementary observational evidence regarding carbohydrate intake patterns among recreationally resistance-trained individuals under free-living conditions. Furthermore, the simultaneous assessment of body composition and muscle strength using standardized procedures enabled a comprehensive characterization of participants’ morphofunctional status, while the multivariable analyses allowed the estimation of between-group differences after adjustment for age, height, and relative energy intake.

Nevertheless, this study has several limitations. First, its cross-sectional design precludes causal inference because carbohydrate intake and the study outcomes were assessed within the same study period, preventing the establishment of temporality. Therefore, it cannot be determined whether lower habitual carbohydrate intake contributed to lower adiposity or whether individuals with lower adiposity were more likely to adopt or maintain a lower-carbohydrate dietary pattern. It is also plausible that participants seeking to reduce body fat or maintain a leaner physique intentionally adopted a dietary pattern characterized by lower carbohydrate and energy intake and higher protein intake. Thus, reverse causation represents an alternative explanation for the observed association between carbohydrate intake and adiposity. Selection bias may also have occurred because participants were recruited through convenience sampling from a single gym, and those who voluntarily participated may differ from the broader population of resistance-trained individuals in dietary practices, training behaviors, or body composition. Furthermore, survivor bias cannot be excluded, as the study included individuals who had maintained their reported carbohydrate intake pattern for at least three months; individuals who had previously adopted a lower-carbohydrate intake but discontinued it because of poor tolerance, impaired training performance, or other unfavorable experiences may therefore not have been represented. These potential sources of bias may have influenced the observed associations and further limit their causal interpretation and generalizability.

Second, dietary intake was assessed using three non-consecutive 24 h dietary recalls collected within a single week. Although the use of multiple non-consecutive recalls, including a weekend day or public holiday, was intended to capture day-to-day variation in intake, these measurements primarily reflect recent dietary intake and cannot independently establish that the observed carbohydrate intake represented participants’ habitual intake over the preceding three months. The ≥3-month duration of the reported dietary pattern was based on participant self-report and was not validated using objective biomarkers or repeated dietary assessments over the entire exposure period. Therefore, recall bias, underreporting, within-person dietary variability, and misclassification of habitual carbohydrate exposure cannot be excluded, and the classification of participants as having low or normal habitual carbohydrate intake should be interpreted with caution.

Third, the a priori sample size calculation was based on a large effect size derived from an experimental study comparing ketogenic and conventional diets, which may not be directly generalizable to the differences in carbohydrate exposure expected under observational, free-living conditions. Although the final sample exceeded the minimum calculated sample size, the study may still have had limited statistical power to detect smaller between-group differences. Furthermore, body fat mass was considered the primary outcome, whereas the remaining body composition and strength outcomes were secondary, and no formal correction for multiple comparisons was applied. Therefore, findings for secondary outcomes should be interpreted cautiously because multiple statistical comparisons increase the possibility of type I error.

Fourth, residual confounding cannot be excluded. Although the regression models were adjusted for age, height, and relative energy intake, detailed measures of resistance training volume and intensity, such as weekly sets, repetitions, and training loads, as well as the exact duration of previous resistance training experience, were not collected and therefore could not be included as covariates. Eligibility criteria requiring at least one year of continuous resistance training and a minimum training frequency were used to reduce heterogeneity in training status, but these criteria do not fully account for individual differences in training exposure. Protein intake also differed between groups and may represent a potential confounder, particularly for muscle-related outcomes. Given the relatively small sample size, the primary regression models were kept parsimonious to reduce the risk of overfitting. Nevertheless, sensitivity analyses were performed with additional adjustment for relative protein intake. These analyses attenuated the associations with adiposity and yielded significant associations with several strength outcomes, indicating that some findings were sensitive to adjustment for dietary composition. Although no problematic multicollinearity was detected, the changes in the estimates reinforce the possibility of residual confounding and the need for cautious interpretation. Detailed results of these analyses are provided in Supplementary Table S1.

Fifth, body composition was estimated using anthropometric methods rather than criterion methods such as dual-energy X-ray absorptiometry (DXA) or magnetic resonance imaging. In particular, fat mass was derived from adipose mass using the equation proposed by Martin et al. [17], which was developed from cadaver-derived data and may not fully reflect body composition characteristics in living, young, resistance-trained individuals. Therefore, the resulting fat mass estimates may be subject to systematic estimation error and should be interpreted with caution. Biomarkers related to carbohydrate availability, such as muscle glycogen content and circulating ketone body concentrations, substrate oxidation rates, and markers of insulin sensitivity, were not assessed. Likewise, strength assessment was limited to maximal isometric handgrip and knee extension tests. Although these measures were selected because of their feasibility and ease of standardization under field conditions, short-duration maximal isometric contractions rely predominantly on the ATP-PCr system and may therefore be less sensitive to differences in carbohydrate availability than more glycolytically demanding exercise. Dynamic strength, muscular endurance, repeated-set performance, and power output were not assessed; consequently, the present findings regarding maximal isometric strength should not be generalized to these other dimensions of resistance exercise performance.

Finally, the study included only young recreationally resistance-trained men; therefore, the findings cannot be directly generalized to women, elite athletes, or individuals participating in other sporting disciplines.

### 4.3. Future Perspectives

Future longitudinal studies and randomized controlled trials with longer intervention periods are needed to confirm these findings through the controlled manipulation of carbohydrate intake, incorporating objective assessments of muscle glycogen using muscle biopsy or magnetic resonance spectroscopy, circulating ketone bodies, substrate oxidation using indirect calorimetry, markers of insulin sensitivity, and objectively quantified resistance training volume, intensity, and training load. Furthermore, direct comparisons between low habitual carbohydrate intake and structured ketogenic interventions would help determine whether the observed associations are primarily attributable to chronically reduced carbohydrate availability, nutritional ketosis, or other dietary- and training-related factors.

## 5. Conclusions

In conclusion, recreationally resistance-trained young men with a low habitual carbohydrate intake (<3 g/kg/day) exhibited lower adipose mass and fat mass in the primary analyses than those with a normal carbohydrate intake (3–5 g/kg/day), with no clear differences in skeletal muscle mass. These associations with adiposity remained significant after adjustment for age, height, and relative energy intake; however, they were attenuated and no longer statistically significant after additional adjustment for relative protein intake. Conversely, associations between low carbohydrate intake and lower maximal isometric strength emerged in the protein-adjusted sensitivity analyses, while skeletal muscle mass remained unrelated to carbohydrate intake group. These findings indicate that the observed associations with adiposity and strength are sensitive to adjustment for dietary composition and should therefore be interpreted cautiously. Because of the cross-sectional design, causal relationships cannot be established. Future longitudinal studies and randomized controlled trials with longer intervention periods are warranted to confirm these findings and determine whether the observed associations reflect independent relationships between carbohydrate intake, overall dietary composition, body composition, and muscle strength.

## Figures and Tables

**Table 1:** General characteristics, dietary intake, and training frequency of participants according to habitual carbohydrate intake.

Variable	Unit	Normal Carbohydrate Intake (*n* = 25)	Low Carbohydrate Intake (*n* = 28)	
		Mean ± SD/Median [IQR]		*p* Value
Age	years	26.72 ± 6.17	26.21 ± 4.78	0.74 ^a^
Body weight	kg	72.62 ± 7.21	72.27 ± 7.84	0.86 ^a^
Height	cm	173.08 ± 5.84	172.39 ± 5.93	0.67 ^a^
Body mass index	kg/m^2^	24.21 ± 1.74	24.25 ± 1.59	0.93 ^a^
Energy intake	kcal/kg/day	31.51 ± 3.28	25.54 ± 1.73	<0.001 ^a^
Carbohydrate intake	g/kg/day	3.90 [3.40–4.70]	2.45 [2.20–2.70]	<0.001 ^b^
Protein intake	g/kg/day	1.40 [1.40–1.55]	1.70 [1.60–1.70]	<0.001 ^b^
Fat intake	g/kg/day	1.10 [0.90–1.20]	1.10 [0.90–1.10]	0.27 ^b^
	Category	n(%)		
Training frequency	Every other day	21(84)	27(96.4)	0.17 ^c^
	Daily	4(16)	1(3.6)	

Note. Data are presented as mean ± standard deviation (SD), median [interquartile range (IQR)], or frequency (%), as appropriate. ^a^ Welch’s *t*-test; ^b^ Mann–Whitney U test; ^c^ Fisher’s exact test. Variables analyzed using Welch’s *t*-test are presented as mean ± SD, whereas variables analyzed using the Mann–Whitney U test are presented as median [IQR]. No transformations of the original variables were performed. A two-sided *p* value < 0.05 was considered statistically significant.

**Table 2:** Body composition and muscle strength according to habitual carbohydrate intake.

Variable	Unit	Normal Carbohydrate Intake (*n* = 25)	Low Carbohydrate Intake (*n* = 28)			
		Mean ± SD/Median [IQR]		Between-Group Difference (95% CI)	*p* Value	Effect Size
Adipose mass	kg	17.17 ± 3.00	14.75 ± 2.44	2.42 (0.90 to 3.94)	<0.01 ^a^	0.88
Adipose mass	%	23.70 ± 3.74	20.45 ± 2.71	3.24 (1.42 to 5.07)	<0.001 ^a^	0.99
Fat mass	kg	10.76 ± 2.49	8.64 ± 1.83	2.12 (0.90 to 3.34)	0.001 ^a^	0.97
Body fat	%	14.87 ± 3.33	11.97 ± 2.26	2.90 (1.30 to 4.50)	<0.001 ^a^	1.02
Skeletal muscle mass	kg	33.77 ± 5.91	33.47 ± 4.81	0.30 (−2.70 to 3.30)	0.84 ^a^	0.06
Skeletal muscle mass	%	46.42 ± 6.19	46.37 ± 4.55	0.05 (−2.99 to 3.09)	0.97 ^a^	0.01
Handgrip strength	kg	39.00 ± 7.19	35.75 ± 5.01	3.25 (−0.22 to 6.72)	0.06 ^a^	0.52
Relative handgrip strength	kg/kg	0.51 [0.48–0.57]	0.50 [0.46–0.54]	0.03 (−0.01 to 0.07)	0.12 ^b^	0.24
Isometric knee extension strength	kg	35.00 [32.00–40.50]	33.00 [31.00–35.00]	2.00 (0.00 to 4.00)	0.12 ^b^	0.24
Relative isometric knee extension strength	kg/kg	0.49 [0.45–0.55]	0.45 [0.42–0.50]	0.03 (−0.01 to 0.07	0.06 ^b^	0.29

Note. Normally distributed variables are presented as mean ± standard deviation (SD), whereas non-normally distributed variables are presented as median [interquartile range (IQR)]. ^a^ Welch’s *t*-test; ^b^ Mann–Whitney U test. No transformations of the original variables were performed. Mean differences with 95% confidence intervals (95% CI) and Cohen’s d are reported for variables analyzed using Welch’s *t*-test. For variables analyzed using the Mann–Whitney U test, between-group differences are reported using the Hodges–Lehmann estimator with 95% CI, and rank-biserial correlation (rrb) is reported as the effect size. Positive between-group differences and effect sizes indicate higher values in the normal carbohydrate intake group relative to the low-carbohydrate intake group.

**Table 3:** Adjusted differences in body composition and muscle strength according to habitual carbohydrate intake.

Dependent Variable	Adjusted β	SE	95% CI	*p* Value	Adjusted R^2^
Adipose mass, kg	−2.42	1.02	−4.49 to −0.35	0.02	0.348
Adipose mass, %	−4.64	1.40	−7.47 to −1.80	<0.01	0.175
Fat mass, kg	−2.33	0.87	−4.08 to −0.57	0.01	0.288
Body fat, %	−4.06	1.22	−6.52 to −1.59	<0.01	0.182
Skeletal muscle mass, kg	2.62	2.07	−1.56 to 6.80	0.21	0.178
Skeletal muscle mass, %	1.25	2.36	−3.51 to 6.00	0.60	−0.056
Handgrip strength, kg	−0.59	1.71	−4.04 to 2.85	0.73	0.602
Relative handgrip strength, kg/kg	−0.032	0.02	−0.079 to 0.015	0.18	0.531
Isometric knee extension strength, kg	−1.28	1.44	−4.18 to 1.63	0.38	0.563
Relative isometric knee extension strength, kg/kg	−0.041	0.02	−0.082 to −0.001	0.04	0.564

Note. β represents the unstandardized regression coefficient for the group variable, coded as 0 = normal habitual carbohydrate intake and 1 = low habitual carbohydrate intake. Accordingly, β represents the adjusted between-group difference for each outcome, with negative values indicating lower values in the low habitual carbohydrate intake group relative to the normal habitual carbohydrate intake group. SE represents the standard error of the unstandardized regression coefficient. All models were adjusted for age, height, and relative energy intake. Adjusted R^2^ represents the proportion of variance in each outcome explained by the corresponding regression model. No evidence of problematic multicollinearity was observed (VIF: carbohydrate intake group = 2.46, age = 1.11, height = 1.06, and relative energy intake = 2.53; all tolerance values ≥ 0.39).

## Data Availability

The raw data supporting the conclusions of this article will be made available by the authors on request.

## Supplementary Materials

The following supporting information can be downloaded at: https://www.mdpi.com/article/10.3390/jfmk11030351/s1, Table S1: Sensitivity analysis of adjusted differences in body composition and muscle strength according to habitual carbohydrate intake, with additional adjustment for relative protein intake.

## Abbreviations

The following abbreviations are used in this manuscript:

24HR	24-hour dietary recall
RPE	Rating of perceived exertion
SGLT-2	Sodium–glucose cotransporter-2
GLP-1	Glucagon-like peptide-1
ATP	Adenosine triphosphate
ATP-PCr	Adenosine triphosphate–phosphocreatine
