# Supplementary material for: Differences Between Habitual Low and Normal Carbohydrate Intake in Body Composition and Maximal Isometric Strength in Resistance-Trained Men
Source: J Funct Morphol Kinesiol. 2026 Sep 3;11(3):351. doi: 10.3390/jfmk11030351 (PMC13608179; doi:10.3390/jfmk11030351)
Supplement: Supplementary file 1 [file jfmk-11-00351-s001.zip › jfmk-4507666-supplementary.pdf]

Supplementary Table S1. Sensitivity analysis of adjusted differences in body composition and muscle strength according to habitual carbohydrate intake, with additional adjustment for relative protein intake

Dependent variable	Adjusted β	SE	95% CI	p value	Adjusted R^2
Adipose mass, kg	−1.20	1.16	−3.55 to 1.14	0.30	0.38
Adipose mass, %	−2.63	1.56	−5.78 to 0.51	0.09	0.25
Fat mass, kg	−1.30	0.98	−3.29 to 0.69	0.19	0.33
Body fat, %	−2.39	1.37	−5.14 to 0.36	0.08	0.25
Skeletal muscle mass, kg	−0.55	2.28	−5.15 to 4.03	0.80	0.27
Skeletal muscle mass, %	−2.93	2.53	−8.03 to 2.15	0.25	0.11
Handgrip strength, kg	−4.21	1.74	−7.72 to −0.71	0.01	0.69
Relative handgrip strength, kg/kg	−0.07	0.02	−0.12 to −0.02	<0.01	0.62
Knee extension strength, kg	−3.77	1.55	−6.90 to −0.65	0.01	0.63
Relative knee extension strength, kg/kg	−0.07	0.02	−0.11 to −0.02	<0.01	0.60

Note: β represents the unstandardized regression coefficient for the carbohydrate intake group, coded as 0 = normal habitual carbohydrate intake and 1 = low habitual carbohydrate intake. Accordingly, β represents the adjusted between-group difference for each outcome, with negative values indicating lower values in the low habitual carbohydrate intake group relative to the normal habitual carbohydrate intake group. SE represents the standard error of the unstandardized regression coefficient. All models were adjusted for age, height, relative energy intake (kcal/kg/day), and relative protein intake (g/kg/day). Adjusted R^2 represents the proportion of variance in each outcome explained by the corresponding regression model. Multicollinearity was assessed using variance inflation factor (VIF) and tolerance values. No evidence of problematic multicollinearity was observed (VIF: carbohydrate intake group = 3.35, age = 1.11, height = 1.07, relative energy intake = 2.59, and relative protein intake = 1.60; all tolerance values ≥ 0.29).
